# S100A7 enhances invasion of human breast cancer MDA-MB-468 cells through activation of nuclear factor-κB signaling

**DOI:** 10.1186/1477-7819-11-93

**Published:** 2013-04-23

**Authors:** Huamin Liu, Lei Wang, Xingang Wang, Zhiwei Cao, Qifeng Yang, Kejun Zhang

**Affiliations:** 1 Department of Oncology, the Affiliated Hospital of Medical College, Qingdao University, Qingdao, Shandong 266003, R.P. China; 2 Department of Thyroid surgery, the Affiliated Hospital of Medical College, Qingdao University, Qingdao, Shandong 266003, R.P. China; 3 Department of Breast surgery, Medical College, Shandong University, Jinan 250014, People’s Republic of China

**Keywords:** Breast cancer, Invasion, S100A7,NF-κB

## Abstract

**Background:**

S100A7 signaling plays a critical role in the pathogenesis and progression of human breast cancers but the precise role and mechanism of S100A7 for tumor invasion remains unclear. in the present study, we investigated whether S100A7 overexpression could be mechanistically associated with the up-regulation of NF-κB, VEGF and MMP-9, resulting in the promotion of breast cancer cell invasion and growth, and *vice versa*.

**Methods:**

pcDNA3.1-S100A7 cDNA plasmid was constructed and transfected into the MDA-MB-468 cells. 4,5-dimethythiazol-2-yl)-2,5-diphenyl tetrazolium bromide (MTT) assay was used to detect cell proliferation, Matrigel was used to detect cell mobility and invasion in vitro.The MMP-9 and VEGF expression and levels was detected by western blot and ELISA assay. NF-κB DNA binding activity was detected by Electrophoretic mobility shift assay.

**Results:**

Up-regulation of S100A7 by stable S100A7 cDNA transfection increased cell invasion and proliferation, whereas downregulation of S100A7 by small interfering RNA in S100A7 cDNA-transfected MDA-MB-468 cells decreased cell invasion and proliferation. Consistent with these results, we found that the up-regulation of S100A7 increased NF-κB DNA-binding activity and MMP-9 and VEGF expression. Down-regulation of S100A7 in S100A7 cDNA -transfected decreased NF-κB DNA-binding activity and MMP-9 and VEGF expression.

**Conclusions:**

Our data demonstrate that the S100A7 gene controls the proliferation and invasive potential of human MDA-MB-468 cells through regulation of NF-κB activity and its target genes, such as MMP-9 and VEGF expression. Down-regulation of S100A7 could be an effective approach for the down-regulation and inactivation of NF-κB and its target genes, such as MMP-9 and VEGF expression, resulting in the inhibition of invasion and growth.

## Background

Human psoriasin (S100A7) is a small calcium binding protein, which has been shown to be predominantly expressed in high-grade ductal carcinoma *in situ*[1-5]. In addition, its expression is significantly associated with estrogen receptor (ER) α-negative and nodal metastasis in invasive ductal tumors [1,3-5]. Furthermore, S100A7 expression is associated with increased angiogenesis [6]. S100A7 has been shown to modulate tumor growth by activating several signaling pathways [7-9]. Recently, it has reported that S100A7 enhances breast tumor growth and metastasis in MDA-MB-468 ERα (−) cells by activating proinflammatory and metastatic pathways [10].

Nuclear factor-kappa B (NF-κB) is an essential transcription factor that not only modulates cellular responses to stress but also plays a pivotal role in inflammation, immunity, cell cycle growth and survival. NF-κB regulated genes have been documented to be involved in cellular proliferation and invasion along with tumor related angiogenesis and lymph angiogenesis [11-13]. Dysregulation of NF-κB associated pathways are seen in multiple malignancies. Its constitutive activation in the clinically aggressive and prognostically poor ER-negative, Her2-neu positive and inflammatory breast cancer could form the basis for its evolution as a potential prognostic and therapeutic target [14].

In MDA-MB-468 cells, inhibition of NF-κB by adenovirus-mediated expression of a dominant negative NF-κB or by a proteasome inhibitor, MG132, decreased the vascular endothelial growth factor (VEGF) mRNA and prevented angiogenesis [15].

In addition, targeting NF-κB signaling may also inhibit breast cancer cell invasion through decreasing matrix metalloproteinase 9 (MMP-9) expression [16]. Emberley *et al*. [17] has recently reported that overexpression of S100A7 in MDA-MB-468 cell lines promotes survival under conditions of anchorage-independent growth. This effect is paralleled in part by increased activity of NF-κB, which is known to mediate prosurvival pathways. The data suggested that there is significant correlation between S100A7 and NF-κB activity.

S100A7 is among the most highly expressed genes in preinvasive breast cancer, is a marker of poor survival when expressed in invasive disease, and promotes breast tumor progression in experimental models [1-3,5,10]. However, it has not been established whether S100A7 may be a target for breast cancer therapy. In the present study, we explored the effect and mechanism of S100A7 silencing on growth and invasion in MDA-MB-468 cell lines.

## Methods

### Cell culture and reagents

MDA-MB-468 cells were obtained from American Type Culture Collection (ATCC, Shanghai Bioleaf Biotech Co., Ltd. Shanghai, China). The MDA-MB-468 cells were grown in (D)MEM with 10% fetal bovine serum and 1% penicillin/streptomycin. The cells were grown in a humidified atmosphere of 5% CO2 at 37°C. Cells were seeded in 75 cm^2^ flasks with 15 ml of growth medium, unless otherwise mentioned. Antibodies were obtained as follows: anti-S100A7, MMP-9, VEGF, NF-κB p65 and GAPDH were obtained from Santa Cruz Biotechnology (Santa Cruz, CA, USA). All secondary antibodies were obtained from Pierce (Rockford, IL, USA). S100A7 small interfering RNA (siRNA),VEGF siRNA, MMP-9 siRNA and siRNA control were obtained from Santa Cruz Biotechnology. LipofectAMINE 2000 was purchased from Invitrogen (Burlington, ON, Canada).

### Plasmid construction and transfections

cDNA of S100A7 (OriGene Technologies, Shanghai, China) was subcloned into pcDNA3.1. MDA-MB-468 cells were transfected with pcDNA3.1-S100A7 cDNA or pcDNA3.1 using LipofectAMINE 2000 reagent according to the manufacturer’s instructions and stable clones were generated using G418 (400 mg/mL)(CAMBREX (Walkersville, MD). Stably transfected MDA-MB-468 cells (pcDNA3.1-S100A7 cDNA/MDA-MB-468 and pcDNA3.1/MDA-MB-468) were transfected with MMP-9 siRNA (2 μM) or VEGF siRNA (2 μM) and control siRNA for 48 hours using LipofectAMINE 2000.

### Electrophoretic mobility shift assay

Nuclear extracts were prepared from cells using the NE-PER nuclear and cytoplasmic extraction reagents kits according to the manufacturer’s directions (Pierce Biotech). For NF-κB DNA binding, the 10,000 cpm of the 22-bp oligonucleotide 5′-AGTTGAGGGGACTTTCCCAGGC-3′ containing the NF-κB consensus sequence that had been labeled with [−^32^P]ATP (10 mCi/mmol) by T4 polynucleotide kinase was added to 15 μg nuclear extract. The reaction was allowed to proceed for 30 minutes at room temperature. For cold competition experiments, unlabeled NF-κB oligonucleotide as nonspecific competitor gels were dried and directly exposed to a B-1 phosphorimaging screen and visualized with a GS-250 Molecular Imaging System [Bio-Rad (Hercules, CA, USA)]. TATA binding protein (TBP) was used as a nuclear loading control (Abcam Inc. Beijing, China).

### MMP-9 activity assay

MMP-9 activities in whole cell lysates were assessed using the colorimetric Biotrak MMP-9 activity assay (Amersham Biosciences, Shanghai, China) in accordance with the manufacturer’s instructions. Optical densities were quantified using a Vmax microplate spectrophotometer at a wavelength of 405 nm, referenced to 650 nm. Three samples were used for each experimental condition and experiments were performed in triplicate and mean values calculated.

### VEGF assay

The culture medium of the cells in different groups grown in six-well plates was collected. After collection, the medium was spun at 800 × g for 3 minutes at 4°C to remove cell debris. The supernatant was either frozen at −20°C for VEGF assay later or assayed immediately using commercially available ELISA kits (R&D Systems, Inc., Minneapolis, MN, USA).

### Western blot analysis

Cell lysates were prepared from the MDA-MB-468 cells in different conditions and subjected to Western blot analysis using an anti-MMP-9, VEGF, S100A7, NF-κB p65 and an anti-β-actin monoclonal antibody for normalization of protein loading. Immunoreactivity was detected using the ECL chemiluminescence system (Amersham, Piscataway, NJ, USA) and quantified using an imaging densitometer (Model GS-670; Bio-Rad, Hercules, CA, USA).

### Cell proliferation assay

MDA-MB-468 cells were stably transfected with pc. DNA3.1-S100A7 or pc. DNA3.1. Then the cells were seeded at a density of 5 × 10^3^ cells per well on 96-well plates in growth medium supplemented with 10% serum and cultured in a humidified chamber at 37°C for up to 3 days. Viable cells were identified using the 3-(4,5-dimethythiazol-2-yl)-2,5-diphenyl tetrazolium bromide (MTT) assay. Briefly, 200 ul sterile MTT dye (5 mg/ml, Sigma, USA) was added. After 4 hours incubation at 37°C in 5% CO_2_, the MTT medium mixture was removed and 200 ul of dimethyl sulfoxide (DMSO) was added to each well, as was reported. Absorbance was measured at 540 nm using a multi-well spectrophotometer (Thermo Electron, Andover, USA). All experiments were carried out in triplicate.

### Determination of cell mobility

The invasiveness of MDA-MB-468 cells was tested after transfection as previously described. The cells (1 × 10^6^/mL) were added to the upper wells coated with Matrigel (1 mg/mL; Collaborative Research, Inc., Boston, MA, USA) with serum-free medium containing 25 ug/mL fibronectin as a chemoattractive agent in the lower wells. After a 24-hour incubation period, cells that migrated through the filters into the lower chamber were counted by the number of cells on the lower side of the membrane in five random fields after staining with Hema-3 kit(Fisher Scientific (Nepean, Canada).

### *In vitro* cell invasion assay using Matrigel

Cell invasion assays were performed using Transwell membrane filter inserts with 8-mm pore size (Corning Costar, Cambridge, MA, USA). The upper surface of the Transwell membrane was coated with 250 mg/ml of growth factor-reduced Matrigel matrix(Becton Dickinson, Bedford, MA, USA) overnight at 4°C, rehydrated once with 0.1% BSA in (D)MEM for 1 hour at room temperature, and then placed in the upper compartment of six-well tissue culture plates.

MDA-MB-468 cells in different conditions were removed from tissue culture flasks by a short exposure to 5 mM ethylenediaminetetraacetic acid (EDTA) and washed once in PBS. Then 2 × 10^5^ cells in serum-free medium containing 0.1% BSA were added to each Transwell chamber and allowed to migrate toward the underside of the membrane for 24 hours in the lower chamber as a chemoattractant. After the cells were fixed in 3.5% paraformaldehyde, cells on the upper surface of the membrane were removed by wiping with a cotton swab, and membranes were mounted onto glass slides. The relative number of invasion was determined by counting the number of invading EGFP(enhanced green fluorescent protein)-positive cells. The number of invading cells transfected with empty vector was assigned a value of 1.0 in each experiment. Twenty random fields/membrane were counted for each assay. Each determination represents the average of three separate experiments.

### Statistical analysis

One-way analysis of variance was used to compare means. The level of statistical significance was set at *P* <0.05, and all statistical calculations were carried out using SPSS.11 software (SPSS Inc., Chicago, IL, USA).

## Results

### Effect of S100A7 overexpression on MDA-MB-468 cells

**Figure 1:**
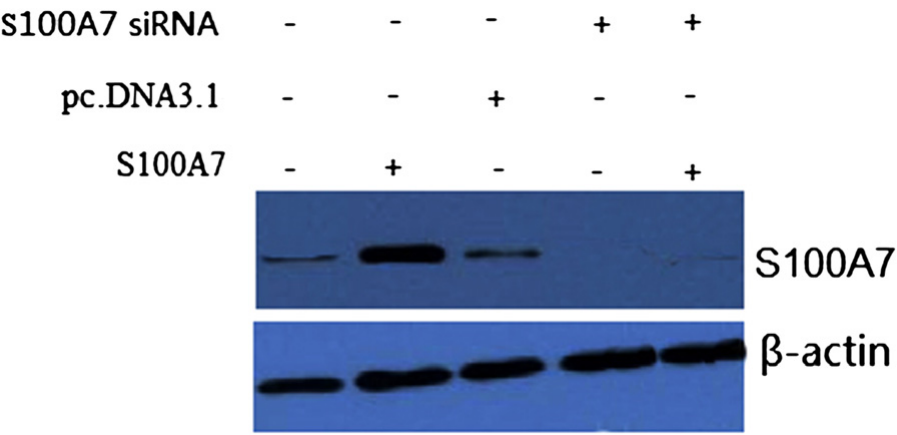
**Effect of S100A7 overexpression by transfection of S100A7-pcDNA3.1 plasmid on S100A7.** Representative images showing expression of S100A7 in vector (pcDNA3.1), pcDNA3.1-S100A7 and transfected with S100A7 siRNA in MDA-MB-468 cells as analyzed by Western blot.

MDA-MB-468 cells stably transfected with pc. DNA3.1-S100A7 plasmid displayed a significant increase in the expression levels of S100A7 as compared with vector control confirmed by performing western blot analysis (Figure 1). When the pc.DNA3.1-S100A7/MDA-MB-468 cells were transfected with S100A7 siRNA for 48 hours,S100A7 expression levels were significantly decreased (Figure 1).

### Effect of S100A7 overexpression on invasive capability

**Figure 2:**
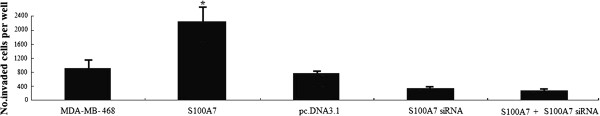
**Effect of S100A7 overexpression on invasiveness of MDA-MB-468 cells transfected with recombinants in Matrigel invasion assay.** Cells were stably transfected with empty vector pc.DNA3.1 or pc.DNA3.1-S100A7, or transfected with S100A7 siRNA. After 2 days, 1 × 10^5^ cells were allowed to invade through transwell inserts (8 μm) coated with Matrigel. The cells on the lower surface of the chambers were stained, counted, and photographed under a light microscope. Histogram shows invasive capability of transfected cells. Each bar represents mean ± SE (n = 3); ^*^, *P* <0.05.

We performed an *in vitro* cell invasion assay using Matrigel matrix to examine whether S100A7 overexpression promotes invasive capability in MDA-MB-468 cells. MDA-MB-468 cells were stably transfected with pc.DNA3.1-S100A7. Repeated experiments revealed that MDA-MB-468/ pc.DNA3.1-S100A7 cells showed significantly higher invasiveness potential than cells transfected with vector alone (Figure 2). However, when S100A7 was silenced by S100A7 siRNA transfection in MDA-MB-468/pc.DNA3.1-S100A7 cells, invasiveness potential was decreased significantly (Figure 2). These results suggest that S100A7enhances the invasiveness of tumorigenic cells.

### Effect of S100A7 overexpression on proliferation

**Figure 3:**
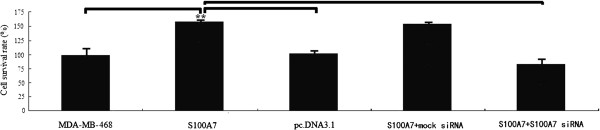
**Effect of S100A7 overexpression on cytotoxicity of breast tumor MDA-MB-468 cell lines.** MDA-MB-468 cells were stably transfected with pc.DNA3.1-S100A7 cells. Cell viability was determined by the MTT assay. The growth rate of the pc.DNA3.1-S100A7 transfected MDA-MB-468 cells was significantly increased in the MTT assay. Each bar represents mean ± SE (n = 3); ^**^, *P* <0.01. MTT, 3-(4,5-dimethythiazol-2-yl)-2,5-diphenyl tetrazolium bromide.

MDA-MB-468 cells were stably transfected with pc.DNA3.1-S100A7 and cell proliferation was detected by MTT analysis. As shown in Figure 3, the growth rate of MDA-MB-468 cells was significantly increased in the pc.DNA3.1-S100A7 transfected cells. However, the growth rate of MDA-MB-468 cells silenced by S100A7 siRNA transfection in pc.DNA3.1-S100A7 stably transfected MDA-MB-468 cells was significant decreased (*P* <0.05), suggesting that S100A7 does alter the cell proliferation rate in MDA-MB-468 cells.

### S100A7 overexpression promotes activation of NF-κB

**Figure 4:**
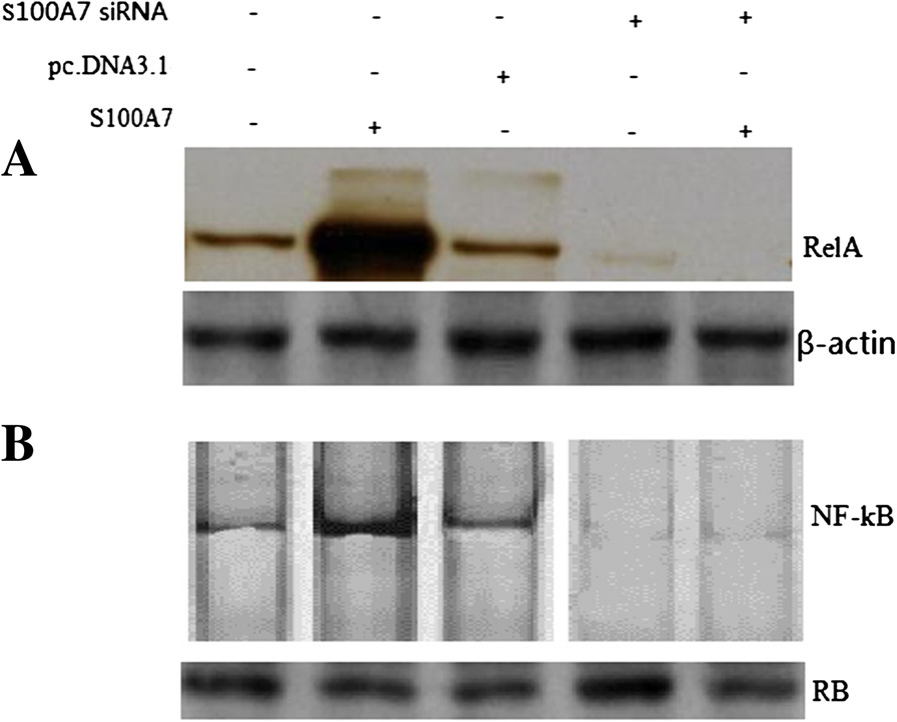
**S100A7 overexpression induced NF-κB in the MDA-MB-468 cells. A**, NF-κB p65 expression in investigated MDA-MB-468 cells was analyzed by western blot. **B**, EMSA analysis was done for MDA-MB-468 cells. Nuclear extracts were prepared from control and transfected cells and subjected to analysis for NF-κB DNA-binding activity as measured by EMSA. Retinoblastoma protein level served as the nuclear protein loading control. EMSA, electrophoretic mobility shift assay; NF-κB, nuclear factor-kappa B.

To investigate whether S100A7 overexpression activated NF-κB in MDA-MB-468 cell lines, western blot was first done to detect NF-κB p65(RelA) levels. Representative pictures are shown in Figure 4A. RelA was overexpressed in MDA-MB-468/pc.DNA3.1-S100A7 cells compared to the control and MDA-MB-468/pc.DNA3.1 cells. The NF-κB signaling pathway is involved in cancer cell invasion processes. Therefore, we measured the NF-κB DNA binding activity in pc.DNA3.1-S100A7 transfected MDA-MB-468 cells. Nuclear extracts from control and pc.DNA3.1 or pc.DNA3.1-S100A7–transfected MDA-MB-468 cells were subjected to analysis for NF-κB DNA-binding activity as measured by electrophoretic mobility shift assay (EMSA). It was found that up-regulation of S100A7 by pc.DNA3.1-S100A7 transfection increased NF-κB DNA-binding activity (Figure 4B). However, S100A7 silencing by S100A7 siRNA transfection significantly induced NF-κB DNA-binding activity in pc.DNA3.1-S100A7 stably transfected MDA-MB-468 cells compared with the control (Figure 4B). These results indicated that S100A7 overexpression increased NF-κB DNA-binding activity in MDA-MB-468 cancer cells. The expression of MMP-9 and VEGF is regulated by NF-κB and has been reported to play an important role in tumor invasion. We, therefore, investigated whether MMP-9 and VEGF were involved in invasion induced by S100A7.

### Effect of S100A7 overexpression on MMP-9 expression and its activity

**Figure 5:**
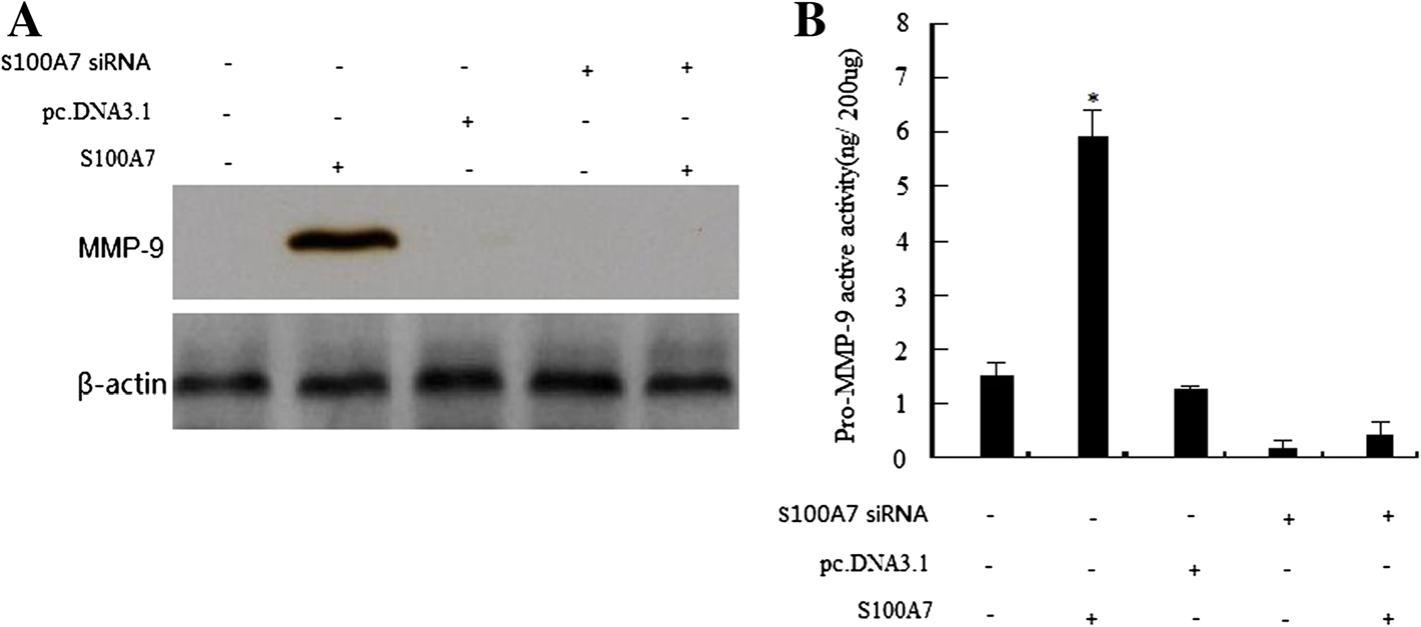
**MMP-9 expression was up-regulated by S100A7 cDNA transfection and down-regulated by S100A7 siRNA transfection. A**, Western blot analysis of MMP-9 protein expression in transfected MDA-MB-468 cells. **B**, MMP-9 activity assay showing that MMP-9 was up-regulated by S100A7 cDNA transfection and down-regulated by S100A7 siRNA transfection. Each bar represents mean ± SE (n = 3); ^*^, *P* <0.01. MMP-9, matrix metalloproteinase 9.

To explore whether the invasiveness of transfected cells was associated with MMP-9 induction, Western blotting was conducted to detect the alteration in the expression of MMP-9. We found that MDA-MB-468 cells transfected with pc.DNA3.1-S100A7 exhibited a significant increase in the expression level of MMP-9 (Figure 5A). However, S100A7 silencing by S100A7 siRNA transfection significantly decreased MMP-9 in pc.DNA3.1-S100A7 stably transfected MDA-MB-468 cells compared with the control (Figure 5A). Next, we examined whether S100A7 overexpression could lead to an increase in MMP-9 activity. There was a four-fold increase in the activity of MMP-9 (Figure 5B). Furthermore, we found that S100A7 silencing decreased the activity of MMP-9 (Figure 5B).

### Effect of S100A7 overexpression on VEGF expression and its activity

**Figure 6:**
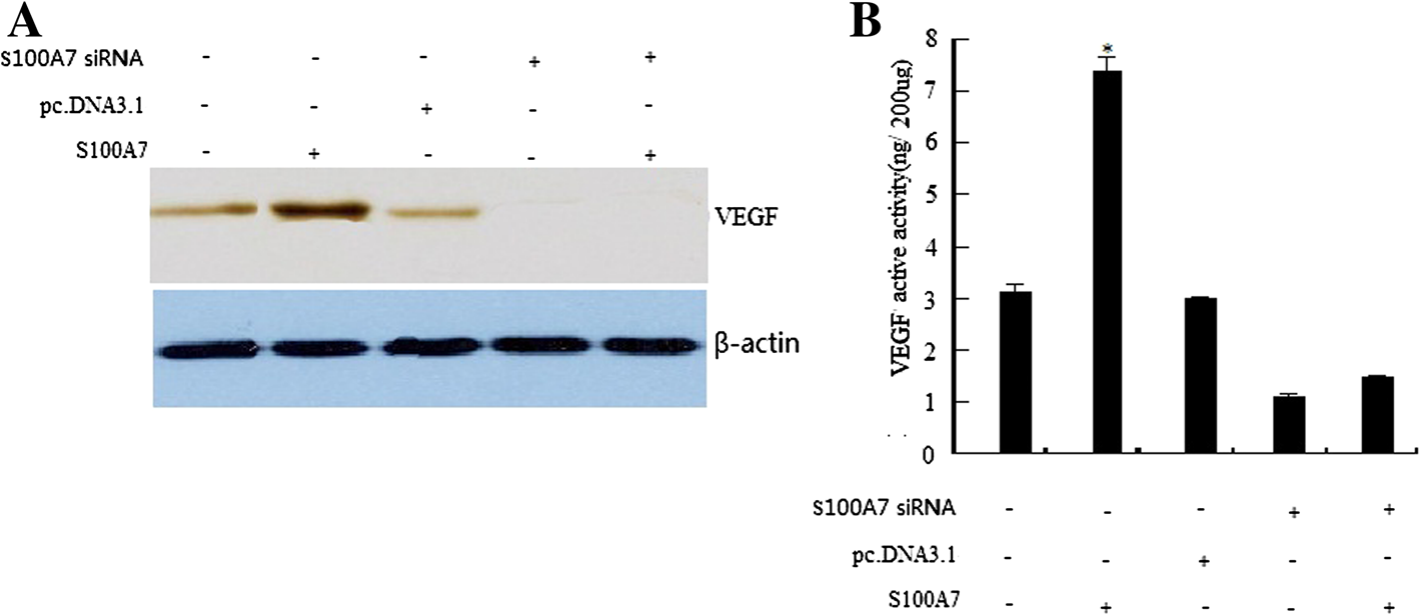
**VEGF expression was up-regulated by S100A7 cDNA transfection and down-regulated by S100A7 siRNA transfection. A**, Western blot analysis of VEGF protein expression in transfected MDA-MB-468 cells. **B**, VEGF activity assay showing that VEGF was up-regulated by S100A7 cDNA transfection and down-regulated by S100A7 siRNA transfection. Each bar represents mean ± SE (n = 3); ^*^, *P* <0.05. VEGF, vascular endothelial growth factor.

We further investigated whether S100A7 expression has any effect on VEGF induction, whose expression is transcriptionally regulated by NF-κB. Western blotting was done to detect the expression of VEGF. We found that VEGF protein levels were dramatically increased in the pc.DNA3.1-S100A7-transfected MDA-MB-468 cells (Figure 6A). Most importantly, we also found that the downregulation of S100A7 could lead to a decrease in VEGF level in pc.DNA3.1-S100A7 stably transfected MDA-MB-468 cells (Figure 6B). Our results also showed that overexpression of S100A7 increased the level of VEGF, which is consistent with the regulation of the DNA-binding activity of NF-κB by S100A7.

### Down-regulation of MMP-9 and VEGF decreased cancer cell invasion

**Figure 7:**
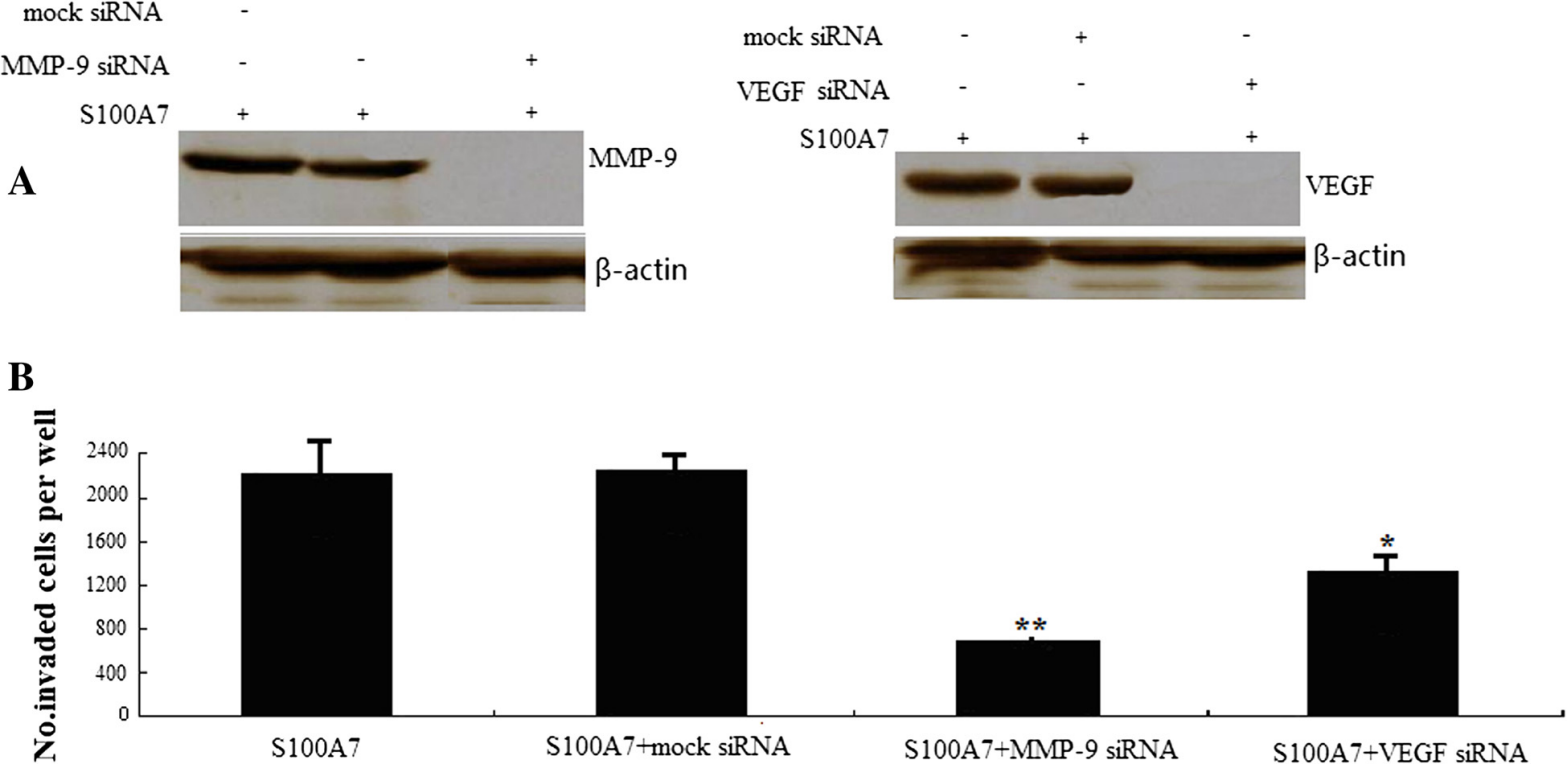
**Down-regulation of MMP-9 or VEGF by siRNA transfection decreased tumor cell invasion. A**, down-regulation of MMP-9 or VEGF by siRNA transfection showed low-expression of MMP-9 or VEGF protein in S100A7-transfected MDA-MB-468 cells as confirmed by Western blot analysis. **B**, invasion assay showing that MMP-9 or VEGF siRNA–transfected cells exhibited low penetration of cells through the Matrigel-coated membrane, compared with control siRNA-transfected cells. MMP-9, matrix metalloproteinase 9. VEGF, vascular endothelial growth factor.

To further confirm the role of MMP-9 and VEGF in MDA-MB-468 cell invasion, the pc.DNA3.1-S100A7-transfected MDA-MB-468 cells (stable transfectant) were transfected with human MMP-9 siRNA or VEGF siRNA. Down-regulation of MMP-9 or VEGF by siRNA transfection resulted in low expression of MMP-9 or VEGF protein, respectively, as confirmed by Western blot analysis (Figure 7A). To further test whether the S100A7-induced invasion of MDA-MB-468 cells is mediated through MMP-9 and VEGF, we used the Matrigel invasion chamber assay to examine the invasive potential of MMP-9 siRNA- or VEGF siRNA-transfected MDA-MB-468 cells that were stably transfected with S100A7. As illustrated in Figure 7B, both MMP-9 siRNA- and VEGF siRNA–transfected cells showed a low level of penetration through the Matrigel-coated membrane compared with the control siRNA–transfected cells. The value of fluorescence was decreased 4.2-fold and 1.8.-fold in the invaded MDA-MB-468 cells transfected with MMP-9 siRNA and VEGF siRNA, respectively, compared with that of control siRNA (Figure 7B). However, whether MMP-9 or VEGF downregulation modulates S100A7 expression in these cells needs further investigation.

## Discussion

S100A7 is a member of the S100 family of proteins, which have been associated with preinvasive ductal carcinoma in situ (DCIS). Persistent expression of S100A7 occurs in some invasive cancers and is associated with poor prognostic factors [1]. Persistent S100A7 expression also occurs in a subset of invasive breast carcinomas and is linked to worse clinical outcome [5]. S100A7 has been shown to be overexpressed in breast cancers at sites of necrosis in tumor tissues [1,7], as well as in the nasal fluid during allergic inflammatory reactions [17]. Although a number of putative functions have been proposed for S100A7, its biological role, particularly in breast cancer, remains to be defined. S100A7 has been reported to increase NF-κB activity levels in MDA-MB-468 cells [7], and NF-κB-regulated genes have been documented to be involved in cellular proliferation and invasion along with tumor related angiogenesis and lymphangiogenesis [18] but not much is known about the relation between S100A7 and NF-κB as well as its role in NF-κB-induced signaling.

In this study, we characterized the tumor-enhancing effects of S100A7 in MDA-MB-468 breast cancer cells.

We have observed that S100A7 overexpression by cDNA transfection increased tumor cell invasion and promoted proliferation but down-regulation of S100A7 by siRNA decreased cell invasion and inhibited proliferation. S100A7 overexpression increased NF-κB DNA binding activity and NF-κB, MMP-9 and VEGF expression levels and activity, but down-regulation of S100A7 by siRNA decreased NF-κB DNA binding activity and NF-κB, MMP-9 and VEGF expression levels and activity. Taken together, these results further support the view that the downregulation of S100A7 could be an effective approach for the inactivation of NF-κB and down-regulation of its target genes, such as MMP-9 and VEGF expression, resulting in the inhibition of invasion and proliferation.

NF-κB activation has also been reported to be associated with metastatic phenotype and to regulate the expression of a variety of important genes in some cellular responses, including metastasis related genes such as VEGF and MMP-9 [19-27]. Because NF-κB plays important roles in many cellular processes, studies on the interaction of NF-κB activation with other cell signal transduction pathways, including the S100A7 pathway, have received increased attention in recent years. S100A7 has also been reported to crosstalk with the NF-κB pathway [7]. S100A7 strongly induces NF-κB promoter activity and induces NF-κB DNA-binding activity [7]. Activation of NF-κB leads to up-regulation of several downstream target genes, including MMP-9 and VEGF. Thus, the downregulation of S100A7 results in lower NF-κB activity and its downstream targets. Therefore, it is possible that S100A7-induced cell invasion is partly due to activation of the NF-κB pathway.

In the present study, we showed that S100A7 overexpression increased NF-κB expression and NF-κB DNA-binding activity and concomitantly increased the expression and activation of MMP-9 and VEGF. However, downregulation of S100A7 decreased NF-κB expression and NF-κB DNA-binding activity and concomitantly inhibited the expression and activation of MMP-9 and VEGF.

Because we observed that S100A7 overexpression promoted MMP-9 expression, we tested the effects of S100A7 on the invasion of MDA-MB-468 cells. We found that S100A7 overexpression promoted cell invasion of MDA-MB-468 cells, and down-regulation of S100A7 inhibited the cell invasion of MDA-MB-468 cells. These results are consistent with MMP-9 data, showing that down-regulation of S100A7 could inhibit cancer cell invasion partly through downregulation of MMP-9 and *vice versa*.

There was a trend toward an association between expression of VEGF and distant metastasis [28]. In this study, consistent with our invasion data, we found a significant reduction in the expression of VEGF by down-regulation of S100A7 and a significant increase in the expression of VEGF by up-regulation of S100A7. It is well accepted that the expression of MMP-9 and VEGF is regulated by NF-κB [20-28]. Down-regulation of S100A7 inhibited the NF-κB reporter gene and gene products, such as those involved in cell invasion and *vice versa*. Based on our results, we speculate that one possible mechanism by which S100A7 induces invasion is due to activation of NF-κB DNA binding activity, which leads to up-regulation of NF-κB target genes, such as MMP-9 and VEGF.

On the basis of previous results that S100A7 has been shown to enhance tumorigenicity in ERα(−) cells, our study also shows that S100A7 overexpression promoted proliferation; however, S100A7 silencing inhibited proliferation in the MDA-MB-468 cells. On the basis of our results, we propose a hypothetical pathway by which S100A7 may promote cell proliferation of MDA-MB-468 cells partly through the NF-κB signaling pathway. However, further in-depth studies are needed to investigate the precise molecular mechanism regarding the cause and effect relationship between S100A7 and NF-κB.

## Conclusions

We demonstrated that the S100A7 gene controls proliferation and invasion of MDA-MB-468 cells at least in part through the activation of NF-κB and its target genes, such as MMP-9 and VEGF expression, resulting in the inhibition of invasion and proliferation, which could be useful for devising novel preventive and therapeutic strategies for breast cancer. This approach could be realized through development of specific S100A7 inhibitors or use of a gene therapy approach.

## Abbreviations

Bp: Base pair; BSA: Bovine serum albumin; (D)MEM: (Dulbecco’s) modified Eagle’s medium; ELISA: Enzyme-linked immunosorbent assay; EMSA: Electrophoretic mobility shift assay; ER: Estrogen receptor; MMP-9: Matrix metalloproteinase 9; MTT: 3-(4,5-dimethythiazol-2-yl)-2,5-diphenyl tetrazolium bromide; NF-κB: Nuclear factor-kappa B; PBS: Phosphate-buffered saline; siRNA: Small interfering RNA; VEGF: Vascular endothelial growth factor.

## Competing interests

The authors declare that they have no competing interests.

## Authors’ contributions

LHM and YQF conceived and designed the study and participated in the part of the experimental work. WL and ZKJ participated in the experimental work and helped draft the manuscript. WXG participated in the analysis and interpretation of the data. CZ and YQF participated in the interpretation of data and revising of the manuscript. All authors read and approved the final manuscript.
